# Independent associations of thyroid-related hormones with hepatic steatosis and insulin resistance in euthyroid overweight/obese Chinese adults

**DOI:** 10.1186/s12876-021-02011-0

**Published:** 2021-11-18

**Authors:** Danyan Ma, Jinyang Zeng, Bingkun Huang, Fangfang Yan, Jiawen Ye, Yun Chen, Xiying Zeng, Xin Zheng, Fangsen Xiao, Mingzhu Lin, Changqin Liu, Zhibin Li

**Affiliations:** 1 School of Medicine, Xiamen University, Xiamen, China; 2 Department of Endocrinology and Diabetes, The First Affiliated Hospital, Xiamen University, Xiamen, China; 3 Xiamen Clinical Medical Center for Endocrine and Metabolic Diseases, Xiamen, China; 4 The Third Clinical Medical College, Fujian Medical University, Fuzhou, China; 5 Fujian Province Key Laboratory of Diabetes Translational Medicine, Xiamen, China; 6 Epidemiology Research Unit, Translational Medical Research Center, The First Affiliated Hospital, Xiamen University, Xiamen, China

**Keywords:** Hepatic steatosis, Insulin resistance, Free triiodothyronine (FT3), Controlled attenuation parameter (CAP), Fatty liver index (FLI)

## Abstract

**Purpose:**

The aim of the study is to explore the independent association of free triiodothyronine (FT3), free thyroxine (FT4) and thyroid stimulating hormone (TSH) with hepatic steatosis and insulin resistance.

**Methods:**

A cross-sectional study of 88 overweight/obese adults who underwent anthropometric measurements [BMI, waist circumference (WC) and waist-to-height ratio (WHtR)], hepatic steatosis assessment (FibroScan) and thyroid-related hormones tests was conducted from 2018 to 2020 in Xiamen, China.

**Results:**

Subjects with increasing tertiles of FT3 showed significantly higher levels of controlled attenuation parameter (CAP) ((295.4 ± 44.1, 290.1 ± 68.2 and 331.7 ± 43.6 (dB/m) for tertile 1–3, respectively, *p* = 0.007) and fatty liver index (FLI) score (47.7 (33.9–60.8), 61.5 (45.1–88.9) and 90.5 (84.5–94.8), respectively, *p* < 0.001). FT3 significantly and positively correlated with obesity index (BMI, WC, and WHtR), homeostatic model assessment of insulin resistance (HOMA-IR) and hepatic steatosis (CAP and FLI). Multivariable linear regression analyses with adjustment for potential confounding factors showed FT3 was independently associated with BMI (regression coefficient (β (95%CI): 0.024 (0.004–0.043), *p* = 0.020), HOMA-IR (β (95%CI): 0.091 (0.007–0.174), *p* = 0.034), CAP (β (95%CI): 25.45 (2.59–48.31), *p* = 0.030) and FLI (β (95%CI): 0.121 (0.049–0.194), *p* = 0.001). Neither FT4 nor TSH was significantly associated with any indicators of obesity, insulin resistance or hepatic steatosis.

**Conclusions:**

Increased FT3, but not FT4 or TSH, was independently associated with higher risks of hepatic steatosis and insulin resistance in euthyroid overweight/obese Chinese adults.

*Trial registration* Registration is not applicable for our study.

## Introduction

Obesity is a worldwide health problem which leads to a series of metabolic disorders via mechanisms of insulin resistance. Nonalcoholic fatty liver disease (NAFLD) as one of metabolic disorders ranges from simple steatosis to nonalcoholic steatohepatitis with fibrosis, which will eventually develop into cirrhosis and hepatocellular carcinoma and is closely related to extrahepatic complication such as dyslipidemia, cardiovascular disease, chronic kidney disease, obstructive sleep apnea syndrome and type 2 diabetes (T2D) [1]. Studies have shown that the prevalence of NAFLD in Asia is around 25% [2], and the value will be higher in obese people [3]. Besides, dysfunctional adipose tissue in obese people is closely related to inflammation and insulin resistance (IR) which lead to the occurrence of type 2 diabetes [4].

Liver biopsy is the gold standard for diagnosing of NAFLD but is expensive and invasive, and cannot be easily adopted worldwide. Thus, several noninvasive imaging methods have emerged such as ultrasonography, computerized tomography (CT), and magnetic resonance imaging (MRI) [5]. However, ultrasonography is subjective and susceptible to many factors. CT is radioactive and MRI is expensive, both of which are not suitable for everyone. Some anthropometric indices used to assess obesity have been proved to be related with NAFLD. Accumulating evidence has shown that body mass index (BMI), waist-to-hip ratio (WHR) and waist-to-height ratio (WHtR) are useful predictive indicators for the risk of NAFLD [6]. Although the anthropometric measurements are simple and easy to be performed, they are less accurate, operator-dependent and cannot be used to assess the severity of NAFLD. The controlled attenuation parameter (CAP), a novel ultrasound-based technique for measuring fat content in the liver could make up for the above shortcomings [7]. CAP is a promising point-of-care technique which could be used to rapidly and non-invasively assess hepatic steatosis [8]. Furthermore, The Fatty Liver Index (FLI) based on BMI, waist circumference (WC), triglyceride (TG) and gamma-glutamyl-transferase (GGT), as one of several clinical prediction models developed as alternatives for identification of patients with NAFLD, is a simple and accurate predictor of hepatic steatosis in the general population [9].

Thyroid hormones play an important role in the regulation of metabolism, thermogenesis, food intake and fat oxidation. Thyroid dysfunction can lead to obesity and obesity-related complication such as hypertension, dyslipidemia, and IR [10]. And abnormal thyroid function is more common among obese people [11], which may be explained by the increased oxidative stress [12]. There is still controversy about the relationship between different composites of thyroid hormones and NAFLD, with some studies showing that higher free T4 (FT4) levels were associated with lower NAFLD risks and hypothyroidism increased the risk of NAFLD [13], while others demonstrating that free T3 (FT3) levels were positively correlated with NAFLD in euthyroid women [14]. In addition, some researchers have put forward different views from the above that neither FT3 nor FT4 was related to NAFLD [15]. However, most studies were limited to children or the elderly, and thyroid function ranged mostly from subclinical hypothyroidism to the high-normal.

Additionally, thyroid hormones can cause glucose metabolism disorders, increase the blood glucose and lead to diabetes. Several studies have found positive associations between thyroid stimulating hormone (TSH) and IR in hypothyroidism people [16]. However, this association within the normal range of thyroid function is in dispute. Some researchers revealed that homeostasis model of insulin resistance (HOMA-IR) was positively correlated with FT3 and TSH, but negatively related with FT4 [17], while others showed the positive association between FT4 and HOMA-IR [18].

In the present study with 88 overweight/obese Chinese adults, we firstly aimed to explore the independent associations of different composites of thyroid-related hormones (FT3, FT4 and TSH) with two indicators of hepatic steatosis (CAP and FLI). Secondly, we also aimed to determine the different association of FT3, FT4 and TSH with insulin resistance which plays the key role linking obesity with metabolic disorders.

## Methods

### Participants

A total of 101 overweight/obese participants recruited into this study from November 2018 to October 2020 in the Department of Endocrinology and Diabetes, the First Affiliated Hospital of Xiamen University, Xiamen, China. Overweight/obese participants (defined as below) who aged from 18 to 50 years were eligible. Exclusion criteria for the present study were long-term drinking history, thyroid dysfunction, increased cortisol, and presence of known liver disease such as viral or autoimmune hepatitis, and treatment of hepatotoxic medications. A face-to-face interview by using standardized questionnaire was conducted for each participant to collect their living habits, disease history and medicine history. Finally, 13 individuals without thyroid hormone related data were excluded and 88 participants were kept in the present analysis. The study was approved by the Human Research Ethics Committee of the First Affiliated Hospital of Xiamen University (Xiamen, China). Written informed consent was obtained from each participant.

### Anthropometric and laboratory measurements

Anthropometric measurements were conducted as described in detail previously [19]. Subjects underwent weight, height, and WC measurements by using a calibrated scale after removing shoes and heavy clothes. BMI was calculated as the weight in kilograms divided by the square of the height in meters. And overweight and obesity were defined as BMI of 24–27.9 kg/m^2^ and ≥ 28 kg/m^2^, respectively [20]. WHtR was calculated as the WC in meters divided by the height in meters. Arterial blood pressure was measured with OMRON electronic sphygmomanometer after sitting for at least 15 min. Blood samples were obtained after 12-h fasting for each subject. Lipid profiles (TG, total cholesterol (TC), and high-density lipoprotein cholesterol (HDL-c)) were determined on a HITACHI 7450 analyzer (HITACHI, Tokyo, Japan). Low-density lipoprotein cholesterol (LDL-C) was calculated by Friedewald’s formula. Fasting plasma glucose (FPG) were measured by the hexokinase method. Serum fasting insulin concentration was measured by electrochemiluminescence immunoassay (Roche Elecsys Insulin Test, Roche Diagnostics, Mannheim, Germany). HOMA-IR was calculated using the formula: fasting serum insulin (mU/L) *fasting plasma glucose (mmol/L) /22.5 [21]. FT3, FT4 and TSH levels were measured using electrochemiluminescence immunoassay.

### Hepatic steatosis assessment

In order to diagnose and assess the severity of hepatic steatosis, CAP was performed using FibroScan® (Echosens, Paris, France) by experienced operators [22]. If transient elastography failed to take ten successful shots, the CAP measurement would be considered invalid [22]. FLI score was calculated using the formula: FLI = e^y^/ (1 + e^y^) * 100, where y = 0.953 * ln (triglycerides, mg/dl) + 0.139 * BMI (kg/m^2^) + 0.718 * ln(GGT, U/L) + 0.053 * waist circumference (cm) – 15.745 [9].

### Statistical analysis

Data were presented as the mean ± standard deviation (SD) or as median (inter-quartile range) for continuous variables and number (proportions) for categorical variables. All subjects were stratified by the tertile of FT3. Differences between the three groups were analyzed on continuous variables using one-way ANOVA for those with normal distributions and Kruskal–Wallis test for those with skewed distributions and on categorical variables using chi-square test.

Pearson’s correlation analyses were performed to explore the correlation coefficients between thyroid hormones (FT3, FT4 and TSH) with anthropometric and biochemical features as well as indicators of hepatic steatosis. Multivariable linear regression analyses were performed to explore the independent associations of different composites of thyroid hormones with obesity indices, hepatic steatosis (CAP and FLI) and HOMA-IR as well. BMI, FLI and HOMA-IR did not follow normal distributions and were log-transformed to obtain better approximation of normal distributions. In model 1, no variable was adjusted for. While in model 2, age, sex, occasional drinking, systolic blood pressure (SBP), diastolic blood pressure (DBP) and TSH were adjusted for; and TG, TC, HDL-c and LDL-c were further adjusted in model 3. All p-values were two-sided and *p*-value < 0.05 was considered statistically significant. All analyses were performed with SPSS version 21.0 software (IBM Corporation).

## Results

Of the 88 overweight/obese adults, 24 (27.3%) were male; and the mean (± SD) of age was 30.0 ± 7.2 years old and their median of BMI was 30.0 (27.7–33.4) kg/m^2^. In terms of dimensions, the means of WC and WHtR were 97.2 ± 11.8 cm and 0.59 ± 0.06, respectively. For thyroid hormones, the mean of FT4 was 17.06 ± 2.53 pmol/L, and the medians of FT3 and TSH were 5.25 (4.87–5.61) pmol/L and 2.24 (1.48–2.98) mIU/L, respectively. In addition, for the indexes of NAFLD, the mean of CAP was 305.0 ± 55.0db/m, and the median of FLI was 69.3 (46.0–90.9).

### Clinical characteristics of subjects by tertiles of serum FT3 level

**Fig. 1:**
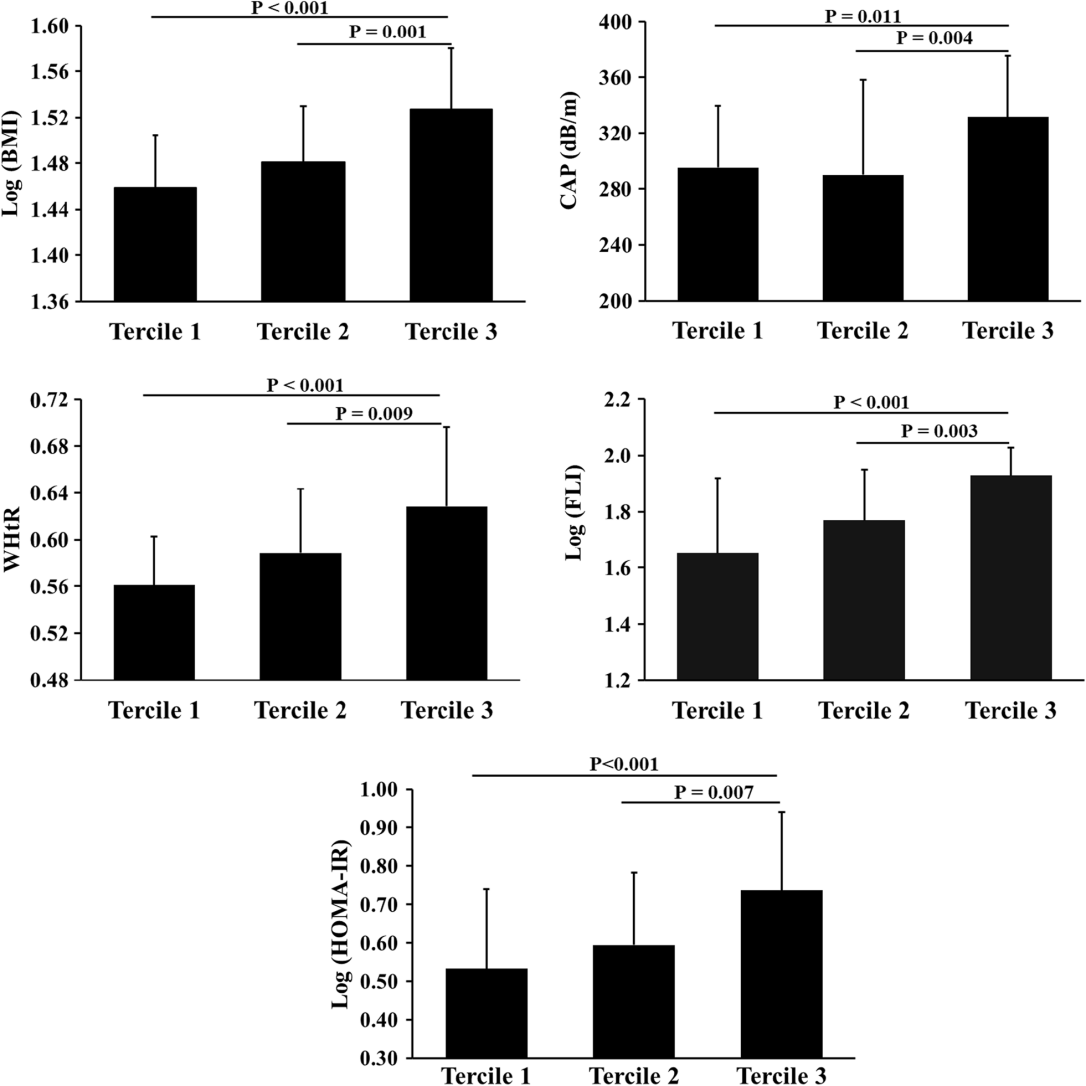
Distributions of BMI(log-transformed), WHtR, CAP, FLI and HOMA-IR (log-transformed) stratified by FT3 tertiles

Subjects were categorized as three groups based on tertiles of serum FT3 levels (median (inter-quartile range): 4.76 (4.61–4.87), 5.15 (5.10–5.28) and 5.95 (5.58–6.36) pmol/L, respectively). Table 1 shows that, with increasing levels of serum FT3, subjects were more likely to be male and young and had significantly higher levels of BMI, waist circumference, WHtR, systolic BP, fasting insulin, HOMA-IR, FT4 and decreased level of HDL-C. Increased tertiles of serum FT3 were also significantly associated with increased CAP ((295.4 ± 44.1, 290.1 ± 68.2 and 331.7 ± 43.6 (dB/m) for tertile 1–3, respectively, p = 0.007) and FLI (47.7 (33.9–60.8), 61.5 (45.1–88.9) and 90.5 (84.5–94.8), respectively, *p* < 0.001) (Fig. 1). There were no statistically significant differences in the levels of diastolic BP, FPG, TG, TC, LDL-C and TSH among these three groups of serum FT3.

**Table 1:** Characteristics of subjects by tertiles of serum FT3 level

Variables	Tertile 1	Tertile 2	Tertile 3	Total	*P* value
N (%)	29 (33.0%)	30 (34.0%)	29 (33.0%)	88 (100.0%)	
Male gender	2 (6.9%)	5 (16.7%)	17 (58.6%)	24 (27.3%)	< 0.001*
Age (years)	32.2 ± 7.2	29.3 ± 7.4	27.2 ± 5.8	30.0 ± 7.2	0.024*
Ever drinking (n, %)	1 (3.4%)	2 (6.7%)	6 (20.7%)	9 (10.2%)	0.070
BMI (kg/m^2^)	27.9 (26.3–31.3)	29.4 (27.8–32.5)	33.5 (30.6–36.5)	30.0 (27.7–33.4)	< 0.001*
Waist circumference (cm)	91.3 ± 8.4	96.0 ± 10.1	106.1 ± 11.8	97.2 ± 11.8	< 0.001*
WHtR	0.56 ± 0.04	0.59 ± 0.05	0.63 ± 0.07	0.59 ± 0.06	< 0.001*
Systolic blood pressure (mmHg)	118.5 ± 10.8	121.0 ± 11.3	128.3 ± 17.5	122.6 ± 14.0	0.019*
Diastolic blood pressure (mmHg)	80.5 ± 8.7	80.9 ± 9.1	80.9 ± 12.3	80.8 ± 10.0	0.986
FPG (mmol/L)	5.06(4.60–5.42)	4.86(4.61–5.42)	5.14(4.56–5.31)	4.97(4.59–5.35)	0.813
Fasting insulin (pmol/L)	101.8 (82.7–138.2)	120.4 (91.8–158.6)	162.7 (122.8–222.9)	128.3 (91.2–180.9)	0.002*
HOMA-IR	3.25 (2.62–4.55)	3.96 (3.03–5.52)	5.20 (3.83–6.56)	4.03 (3.06–5.85)	0.002*
Triglyceride (mmol/L)	1.36 (1.14–2.26)	1.64 (1.29–2.15)	1.84 (1.58–2.82)	1.63 (1.27–2.54)	0.054
Total cholesterol (mmol/L)	5.27 ± 1.05	4.96 ± 0.84	5.14 ± 0.94	5.14 ± 0.96	0.481
HDL-cholesterol (mmol/L)	1.33 ± 0.29	1.15 ± 0.27	1.23 ± 0.23	1.23 ± 0.27	0.039*
LDL-cholesterol (mmol/L)	3.17 ± 0.74	2.96 ± 0.71	3.08 ± 0.84	3.08 ± 0.78	0.600
FT3 (pmol/L)	4.76 (4.61–4.87)	5.15 (5.10–5.28)	5.95 (5.58–6.36)	5.15 (4.87–5.61)	< 0.001*
FT4 (pmol/L)	15.75 ± 2.41	17.47 ± 2.13	17.94 ± 2.57	17.06 ± 2.53	0.002*
TSH (mIU/L)	2.50 (1.41–3.31)	2.23 (1.52–2.90)	2.09 (1.40–2.80)	2.24 (1.48–2.98)	0.573
FLI score	47.7 (33.9–60.8)	61.5 (45.1–88.9)	90.5 (84.5–94.8)	69.3 (46.0–90.9)	< 0.001*
CAP (dB/m)	295.4 ± 44.1	290.1 ± 68.2	331.7 ± 43.6	305.0 ± 55.0	0.007*

Data were presented as mean ± SD or median (interquartile ranges) for continuous variables, and numbers (proportions) for categorical variables

*SBP* systolic pressure, *DBP* diastolic pressure, *WC* waist circumference, *BMI* body mass index, *WHtR* waist-to-height ratio, *FPG* fasting plasma glucose,* HOMA-IR* homeostatic model assessment of insulin resistance, *TC* total cholesterol, *TG* triglycerides, *HDL-c* high density lipoprotein cholesterol, *LDL-c* low density lipoprotein cholesterol, *FT3* free triodothyronine, *FT4* free thyroxine, *TSH* thyroid stimulating hormone, *FLI* Fatty Liver Index, *CAP* controlled attenuation parameter

*p < 0.05

### Correlations of FT3, FT4 and TSH with clinical characteristics

Table 2 shows the Pearson’s correlation coefficients of serum FT3, FT4 and TSH with clinical indices of obesity, hepatic steatosis, and insulin resistance. For FT3, there were significantly positive correlations with systolic BP, obesity (BMI, WC, and WHtR), fasting insulin, insulin resistance (HOMA-IR) and hepatic steatosis (CAP and FLI) as well as negative correlation with age. FT4 was also significantly and positively correlated with systolic BP, obesity (BMI, WC, and WHtR) and FLI but not with insulin resistance (HOMA-IR) or CAP. TSH was only significantly correlated with FPG but not any other clinical parameters.

**Table 2:** Pearson’s correlation coefficients of FT3, FT4 and TSH with clinical features and hepatic steatosis

Variables	FT3		FT4		TSH	
	r	*p*-value	r	*p*-value	r	*p*-value
Age (years)	−0.271	0.011*	−0.147	0.171	−0.199	0.063
SBP (mmHg)	0.247	0.021*	0.258	0.016*	0.027	0.806
DBP (mmHg)	−0.022	0.841	0.121	0.266	0.095	0.382
WC (cm)	0.487	< 0.001*	0.315	0.003*	0.007	0.946
BMI (kg/m^2^)	0.5	< 0.001*	0.289	0.006*	−0.008	0.938
WHtR	0.405	< 0.001*	0.228	0.035*	0.003	0.979
FPG (mmol/L)	−0.04	0.712	−0.157	0.145	−0.239	0.025*
Fins(pmol/L)	0.376	< 0.001*	−0.002	0.984	0.069	0.526
HOMA−IR	0.366	< 0.001*	−0.034	0.757	−0.021	0.849
TC (mmol/L)	−0.039	0.722	−0.056	0.611	−0.121	0.273
TG (mmol/L)	0.218	0.047*	0.173	0.116	0.014	0.900
HDL-c (mmol/L)	−0.15	0.173	−0.052	0.641	0.132	0.232
LDL-c (mmol/L)	−0.019	0.865	−0.066	0.553	−0.109	0.325
TSH (mIU/L)	−0.081	0.454	−0.158	0.142	−	−
FLI score	0.492	< 0.001*	0.322	0.003*	−0.03	0.785
CAP (dB/m)	0.278	0.009*	0.072	0.504	0.037	0.731

*SBP* systolic pressure, *DBP* diastolic pressure, *WC* waist circumference, *BMI* body mass index, *WHtR* Waist-To-Height Ratio, *FPG* fasting plasma glucose, *Fins* Fasting Insulin, *HOMA-IR* homeostatic model assessment of insulin resistance, *TC* total cholesterol, *TG* triglycerides, *HDL-c* high density lipoprotein cholesterol, *LDL-c* low density lipoprotein cholesterol, *TSH* thyroid stimulating hormone, *FLI* Fatty Liver Index, *CAP* controlled attenuation parameter, *FT3* free triodothyronine, *FT4* free thyroxine, *TSH* thyroid stimulating hormone

*p < 0.05

### Independent associations of FT3, FT4 and TSH with obesity, insulin resistance and hepatic steatosis

Table 3 shows the adjusted linear regression coefficients (β) with associated 95% confidence interval (CI) of serum FT3, FT3 and TSH levels for obesity indices (BMI and WHtR), insulin resistance (HOMA-IR) and hepatic steatosis (CAP and FLI) by using the multivariable linear regression analyses with adjustment for potential confounding factors in three different models. In model 1 and model 2, increasing FT3 was significantly associated with increased risks of obesity (BMI and WHtR), insulin resistance (HOMA-IR) and hepatic steatosis (CAP and FLI). In model 3 with full adjustment, the positive associations of serum FT3 with BMI, HOMA-IR, and hepatic steatosis (CAP and FLI) were still statistically significant, although the association with WHtR became non-significant.

**Table 3:** Multiple linear regression analyses of FT3, FT4 and TSH with obesity indices, hepatic steatosis and HOMA-IR

Variables	BMI (log-transformed)		WHtR		CAP		FLI score (log-transformed)		HOMA-IR (log-transformed)		
	β (95% CI)	*P*-value	β (95% CI)	*P*-value	β (95% CI)	*P*-value	β (95% CI)	*P*-value	β (95% CI)	*P*-value	
**FT3**											
Model 1	0.044 (0.027–0.061)	< 0.001*	0.038 (0.017–0.059)	0.001*	25.11 (6.14–44.07)	0.010*	0.157 (0.083–0.232)	< 0.001*	0.134 (0.064–0.204)	< 0.001*	
Model 2	0.028 (0.009–0.047)	0.005*	0.027 (0.002–0.051)	0.034*	22.36(0.09–44.63)	0.049*	0.093(0.008–0.178)	0.032*	0.09 (0.011–0.17)	0.011*	
Model 3	0.024 (0.004–0.043)	0.020*	0.021 (−0.004–0.046)	0.104	25.45 (2.59–48.31)	0.030*	0.121 (0.049–0.194)	0.001*	0.091 (0.007–0.174)	0.034*	
**FT4**											
Model 1	0.007 (0.002–0.011)	0.004*	0.005 (0.000–0.010)	0.057	1.370 (−3.369–6.108)	0.567	0.015 (−0.005 −0.035)	0.143	−0.002 (−0.020–0.016)	0.841	
Model 2	0.004 (−0.001–0.008)	0.092	0.002 (−0.003–0.007)	0.397	−0.363 (−5.371–4.644)	0.886	0.003 (−0.017–0.022)	0.784	−0.013 (−0.031–0.005)	0.167	
Model 3	0.004 (−0.001–0.008)	0.126	0.002 (−0.004–0.008)	0.485	−1.176 (−6.939–4.587)	0.685	0.007 (−0.011–0.025)	0.436	−0.016 (−0.036–0.004)	0.125	
**TSH**											
Model 1	−0.0004 (−0.009–0.008)	0.929	−0.002 (−0.012–0.007)	0.625	1.655 (−7.107–10.417)	0.708	−0.004 (−0.039–0.032)	0.832	0.001 (−0.033–0.035)	0.962	
Model 2	−0.001 (−0.009–0.006)	0.710	−0.004 (−0.013–0.005)	0.431	2.333 (−6.367–11.032)	0.595	−0.003 (−0.034–0.029)	0.870	−0.003 (−0.035–0.029)	0.868	
Model 3	−0.001 (−0.009–0.007)	0.768	−0.004 (−0.014–0.005)	0.329	3.206 (−6.003–12.415)	0.490	0.003 (−0.026 −0.031)	0.854	0.002 (−0.030–0.035)	0.893	

Model1: NO adjustment

Model2: Adjustment for age, sex, occasional drinking, SBP, DBP and TSH

Model3: Adjustment for age, sex, occasional drinking, SBP, DBP, TSH, TG, TC, HDL-c, and LDL-c

*BMI* body mass index, *WHtR* waist-to-height ratio, *HOMA-IR* homeostatic model assessment of insulin resistance, *FLI* fatty liver index, *CAP* controlled attenuation parameter, *FT3* free triiodothyronine, *FT4* free thyroxine, *TSH* thyroid stimulating hormone

*p < 0.05

In model 3 with adjustment for all potential confounding factors, neither FT4 nor TSH was significantly associated with any indicators of obesity (BMI or WHtR), insulin resistance (HOMA-IR) or hepatic steatosis (CAP and FLI).

## Discussion

In the present study of 88 overweight/obese Chinese adults, we found that increased tertiles of serum FT3 were significantly associated with higher levels of BMI, waist circumference, WHtR, fasting insulin, HOMA-IR, and hepatic steatosis (CAP and FLI). Pearson correlation analyses also showed that FT3 were significantly and positively correlated with the above parameters. With adjustment for potential confounding factors, multivariable linear regression analyses showed that serum FT3 was independently and positively associated with BMI, HOMA-IR, CAP and FLI. However, neither FT4 nor TSH was independently associated with any indicators of obesity, insulin resistance or hepatic steatosis.

Thyroid hormones play essential roles in maintaining metabolic homeostasis, and thyroid dysfunction is now more common among obese population. In the present study, we found that increasing FT3 levels, but not FT4 or TSH, were significantly associated with increased obesity indices (BMI and WHtR), which was consistent with some previous studies [23, 24]. But there were still some different views on this relationship. Du et al. [25] reported an observational study which was conducted in the northernmost region of China and found that no significant association was found between FT3 and components of central obesity (BMI and WHR); but FT4 was negatively, and TSH was positively, correlated with BMI in patients with central obesity. Available evidence as well as ours indicates FT3 plays an important role in the weight regulation. The positive correlation between FT3 and obesity could be explained by the increases of expression and activities of type I iodothyronine 5’-deiodinase in adipose tissue, which was an important source of circulating T3 [26]. Therefore, elevated FT3, a production of adaptation to obesity, could increase energy expenditure to maintain metabolic balance [27].

Thyroid-related hormones achieve the balance between lipid synthesis and lipid oxidation in different ways, and thereby exerts the effect of lowering lipids [28]. A study conducted in Germany including 3661 subjects without self-reported histories of thyroid or liver diseases showed the inverse relationship between FT4 and NAFLD [29]. Tahara et al. [30] found increased TSH within the euthyroid range was an independent risk factor of NAFLD, and might influence the progress of liver fibrosis. Some studies conducted in euthyroid subjects showed that higher level of FT3 was an independent predictor of NAFLD [31] and that FT3 levels changed with the alteration of NAFLD status [14]. Most above studies used liver ultrasound to assess NAFLD which was subjective. In the current study, NAFLD was evaluated by CAP, which was a newly developed non-invasive and quantitative evaluation method and has been widely used as a first-line assessment for screening fatty liver [22]. In addition, CAP values were closely associated with metabolic syndrome (MetS) and its components including obesity, hypertriglyceridemia, hyperglycemia and hypertension [32]. FLI, as is a simple, accurate and non-invasive approach, has also shown a good capability for discriminating individuals with NAFLD from those without it in a population-based study with 7-year follow up [33]. However, there were few studies to explore the association between thyroid-related hormones with CAP and FLI. Our study found that higher FT3, but not FT4 or TSH, was positively associated with CAP and FLI, which was consistent with previous studies [34, 35].

To further explore the potential mechanism of relationship between thyroid-related hormones with obesity and NAFLD, we analyzed the association between thyroid function and IR, which has been implicated in the pathogenesis of both obesity and NAFLD. In our study, we found FT3 was significantly and positively associated with HOMA-IR, which was consistent with two other studies conducted in euthyroid subjects [18, 36]. The possible mechanism may be that FT3, as a biologically active thyroid hormone, can increase the decomposition of glycogen and promote glucose absorption in small intestinal mucosa to increase the production of endogenous glucose, which could convert to lipid and then deposit in the liver at the effect of high level of serum insulin [37–39]. However, there were still different findings on the relationships of FT3 with obesity, NAFLD and IR. Since the inner mechanism underlying NAFLD are far from being clarified [40], the underlying mechanism of relationship between thyroid-related hormones with NAFLD were still not fully understood. One possible reason may be due to the different study populations, such as the difference in age, sex, race, region, and especially the thyroid functions. Many study populations are accompanied by subclinical hypothyroidism or their TSH is in a normal high stage, and some even have hyperthyroidism or hypothyroidism [41, 42]. Subjects in the present study were middle-aged population with normal thyroid function. Therefore, further researches, including more obese and euthyroid people, will be needed to explore the true relationships of thyroid-related hormones with obesity, NAFLD and IR.

Some limitations in the present study should be recognized. First, the sample size was small and all 88 participants were with NAFLD. Therefore, we might under-estimate the true associations of thyroid-related hormones and obesity, NAFLD and IR, and our results should be confirmed in non-obese and non-NAFLD populations. Secondly, hepatic steatosis which made the overall sensitivity and specificity of detection of fatty liver compared to liver biopsy were only 80% to 90%, but not assessed by other modalities such as ratio of liver to spleen (LV/SV ratio) using CT, MRI, and liver biopsy. Thirdly, we cannot determine the temporal sequence between thyroid-related hormones and NAFLD because of the cross-sectional study design. Therefore, a prospective cohort study with larger sample size is needed to address the causal relationships of thyroid-related hormones with hepatic steatosis and IR.

## Conclusion

Elevated FT3, but not FT4 or TSH, was associated with increased risk of hepatic steatosis and IR in euthyroid overweight/obese adults. Closely monitoring the FT3 levels should be addressed in terms of preventing hepatic steatosis and IR-related diseases.

## Data Availability

The data used to support the findings of this study are available from the corresponding author upon reasonable request.

## Abbreviations

NAFLD: Nonalcoholic fatty liver disease
T2D: Type 2 diabetes
IR: Insulin resistance
CT: Computerized tomography
MRI: Magnetic resonance imaging
BMI: Body mass index
WHR: Waist-to-hip ratio
WHtR: Waist-to-height ratio
CAP: Controlled attenuation parameter
FLI: Fatty Liver Index
WC: Waist circumference
TG: Triglyceride
GGT: Gamma-glutamyl-transferase
FT4: Free thyroxine
FT3: Free triiodothyronine
TSH: Thyroid stimulating hormone
HOMA-IR: Homeostasis model of insulin resistance
TC: Total cholesterol
HDL-c: High-density lipoprotein cholesterol
LDL-c: Low-density lipoprotein cholesterol
FPG: Fasting plasma glucose
SD: Standard deviation
SBP: Systolic blood pressure
DBP: Diastolic blood pressure
CI: Confidence interval
MetS: Metabolic syndrome
